# Can haptic reinforced VR simulation transform preclinical pulpotomy training? Insights into skill acquisition, student perceptions, and educational impact: randomized controlled trial

**DOI:** 10.3389/froh.2025.1677056

**Published:** 2025-09-24

**Authors:** Anabel Gramatges-Rojas, María Florencia Sittoni-Pino, Nicla Flacco, Oscar Musalem-Dominguez, Mercedes Spinelli, Szabolcs Felszeghy, Santiago Arias-Herrera

**Affiliations:** 1 Faculty of Health Sciences, Department of Preclinical Dentistry, Universidad Europea de Valencia, Valencia, Spain; 2 School for Doctoral Studies and Research, Universidad Europea de Madrid, Madrid, Spain; 3 School of Medicine, Institute of Biomedicine, University of Eastern Finland, Kuopio, Finland; 4 School of Medicine, Institute of Dentistry, University of Eastern Finland, Kuopio, Finland

**Keywords:** haptic virtual reality, dental education, pediatric dentistry, manual dexterity, preclinical training, pulpotomy

## Abstract

**Introduction:**

Preclinical training in pediatric dentistry is essential for developing the psychomotor and cognitive skills required for procedures such as pulpotomies. Haptic virtual reality simulators (HVRS) are redefining dental training by providing real-time feedback, on-demand objective assessment, and expanded opportunities for skill development—making them powerful complements to traditional mannequin-based methods. However, evidence supporting their effectiveness in pediatric procedures remains limited.

**Methods:**

This randomized controlled study involved 173 third-year dental students who received prior instruction in both HVRS and conventional simulation. Participants were assigned to either a control group (traditional training only) or an experimental group (additional training with the Simodont® HVRS). All students then performed a standardized pulpotomy on a primary molar resin tooth. Performance was assessed across four technical criteria by blinded evaluators. Student perceptions of the HVRS experience were collected through a questionnaire.

**Results:**

No statistically significant differences were found between groups for cavity shape or conservative opening. However, the test group showed significantly higher scores in roof removal (*p* < 0.001), while the control group slightly outperformed in pulp chamber configuration (*p* < 0.033). Overall performance score was slightly higher in the test group, though not statistically significant. Additionally, most students reported that the simulator improved their understanding of the procedure, visual realism, tactile perception, and manual skills, although fewer students felt it enhanced their ability to deroof the pulp chamber. A majority preferred combining haptic simulation with traditional training.

**Conclusion:**

HVRS enhanced specific technical skills in pulpotomy, especially pulp chamber roof removal, and was positively perceived by students as a complementary tool in pediatric preclinical training.

## 1 Introduction

Psychomotor and clinical skills are a great challenge for undergraduate dental students. Preclinical training permits students to develop cognitive and technical abilities as well as self-confidence in a safe and simulated environment before transitioning to patient care (1–3). Traditionally, such training has relied on resin teeth mounted on mannequins or phantom heads. Despite their academical value, these models present important limitations, such as lack of tactile realism, single-use materials, environmental concern, heterogenous instructor feedback, and limited capacity for self-guided learning (2, 3).

Haptic virtual reality simulation (HVRS) combines three-dimensional computer-generated environments with force-feedback technology, enabling users to experience tactile sensations that closely mimic those encountered in real clinical procedures. In dental education, HVRS has been applied to various preclinical and clinical skills training, including cavity preparations, endodontic procedures, and surgical techniques. Compared with conventional mannequin-based training, HVRS offers several potential advantages, such as standardized case scenarios, the possibility of repeated practice without risk to patients, immediate performance feedback, and the opportunity to simulate a wide range of clinical conditions within a controlled environment. These features make HVRS a promising complementary tool in the development of psychomotor skills and clinical decision-making in dentistry (4, 5).

Devices such as Simodont® (Nissin Dental Procedures INC, JPN) have emerged into dental graduate education as a response to those limitations, offering an immersive experience that replicates sensations and provides real-time objective feedback on unlimited performances (6, 7). Some studies have shown that haptic simulation is a valuable tool when related to other instructional methods, enhancing learning outcomes (5), while others observed similar performance outcomes between students trained with and without HVRS (3, 8), however, the majority recognized its benefits and appreciated the learning experience.

Although HVRS has shown promising educational outcomes in various areas of dental education, particularly in restorative Dentistry (9, 10)-there remains a notable gap in evidence regarding its application in pediatric dentistry. In procedures such as pulpotomies, where fine motor control, tissue differentiation, and tactile precision are critical, current studies are scarce (3). Moreover, there is a lack of robust comparative research assessing clinical performance outcomes between students trained with HVRS and those trained through traditional preclinical methodologies. This highlights the need for further studies exploring the effectiveness of HVRS as a complementary tool in pediatric dental education (9, 11).

In this context and considering the increasing integration of immersive technologies in dental education, the present study was conducted to evaluate the potential benefits of incorporating HVRS as a complementary preclinical training tool in pediatric dentistry. Specifically, this study aimed to compare the quality of 8.5 pulpotomy procedure performed by third-year dental students who had undergone prior training with HVRS to those who had not. Additionally, the study assessed students' satisfaction and perceived usefulness of HVRS through a questionnaire. We hypothesized that the integration of HVRS into preclinical pediatric dentistry training for pulpotomy procedures would lead to superior technical performance and higher student satisfaction compared with traditional mannequin-based training alone.

## 2 Materials and methods

### 2.1 Study design

This was a parallel-arm randomized controlled trial with a 1:1 allocation ratio, conducted as a single-blind, prospective 1-month study. The design of this study was performed using the CONSORT Declaration 2010 as a guide for conducting randomized clinical trials of parallel groups (12). The procedural protocol applied to the control and experimental groups, together with the anonymized rubric scores and questionnaire dataset, are provided as Supplementary Materials accompanying this article.

### 2.2 Participants

**Figure 1:**
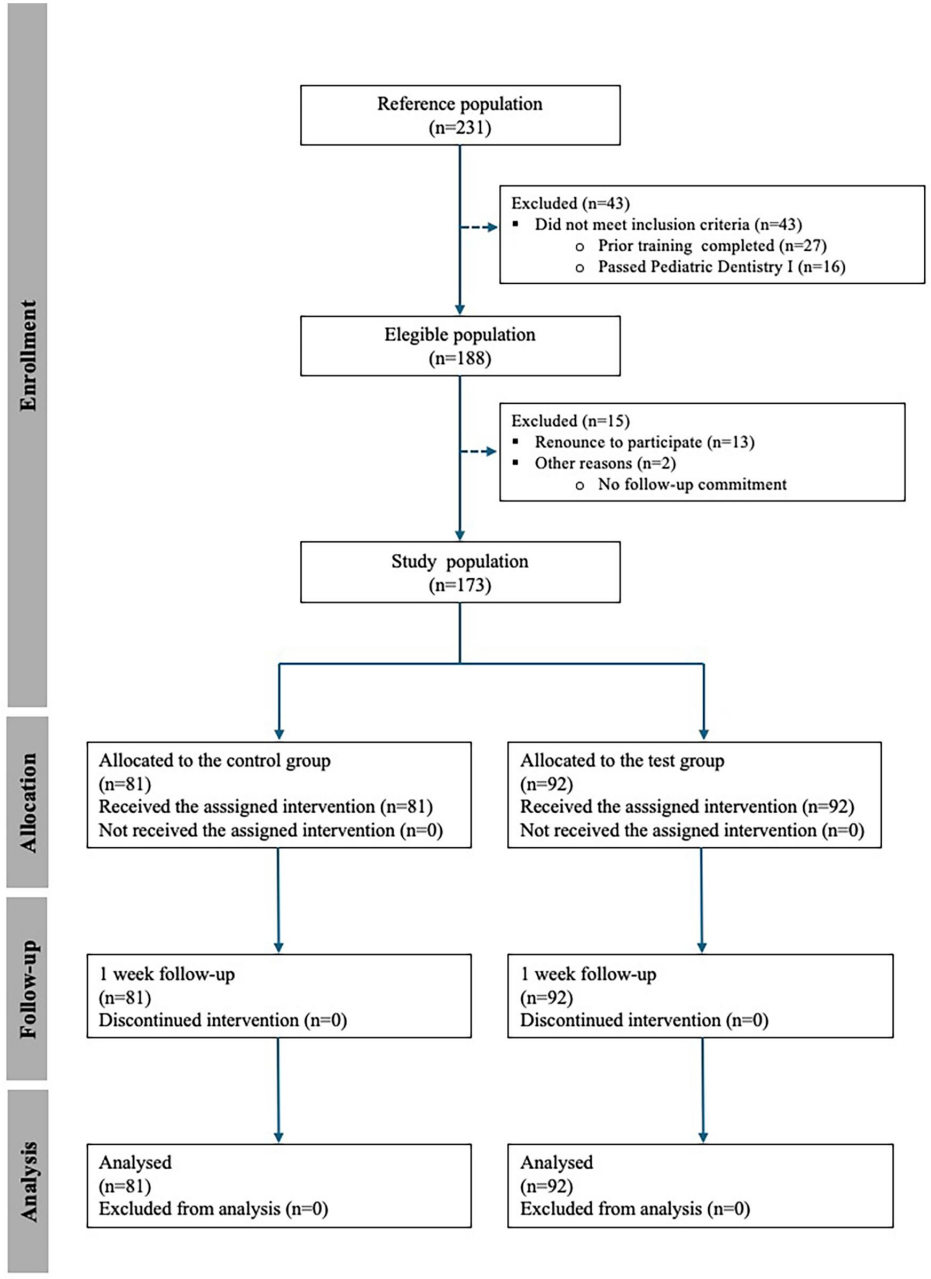
Flow chart of participant selection and allocation throughout the study.

The reference population consisted of third-year students enrolled in Pediatric Dentistry II course during the 2024–2025 academic year at European University of Valencia. The eligible population included those students who fulfilled the inclusion criteria: prior completion of training sessions using both HVRS and traditional phantom head models. Students who had not successfully passed the Pediatric Dentistry I course, which includes foundational preclinical training in cavity preparation, were excluded. These criteria ensured that all participants entered the study with a comparable baseline in both virtual and conventional preclinical training modalities. From this eligible population, the study population was composed of those students who voluntarily agreed to participate and provided informed consent (Figure 1).

### 2.3 Intervention

Students who met the inclusion criteria and voluntarily agreed to participate in the study were randomly assigned into two groups. The control group received conventional preclinical traditional training using child model typodont AK-6/2 from Frasaco® mannequins equipped with primary dentition models. The experimental group received additional training through a HVRS, using the Simodont® dental trainer (Nissin Co., Ltd.) prior to the conventional training.

All participating students had previously been trained in the use of HVRS, having completed a competency-based training course in simulation skills during Pediatric Dentistry I course. This training began with an introductory session in which the Simodont® HVRS was presented, including its components, functionality, and basic usage instructions. Subsequently, students completed a knowledge test to assess their understanding of the simulator. They were allowed unlimited attempts, but only upon successfully passing the test they were permitted to proceed with the practical training.

Likewise, all students had prior experience with conventional preclinical training methods, as they had completed practical exercises in cavity preparation within the Restorative Dentistry I course. This ensured that all participants were familiar with both haptic and traditional simulation environments prior to the intervention, allowing for a more homogeneous baseline in terms of technical exposure and psychomotor skill development.

All students received theoretical instruction on the pulpotomy procedure, followed by a live instructor demonstration and immediate feedback during the hands-on practice.

#### 2.3.1 Haptic virtual reality training

**Figure 2:**
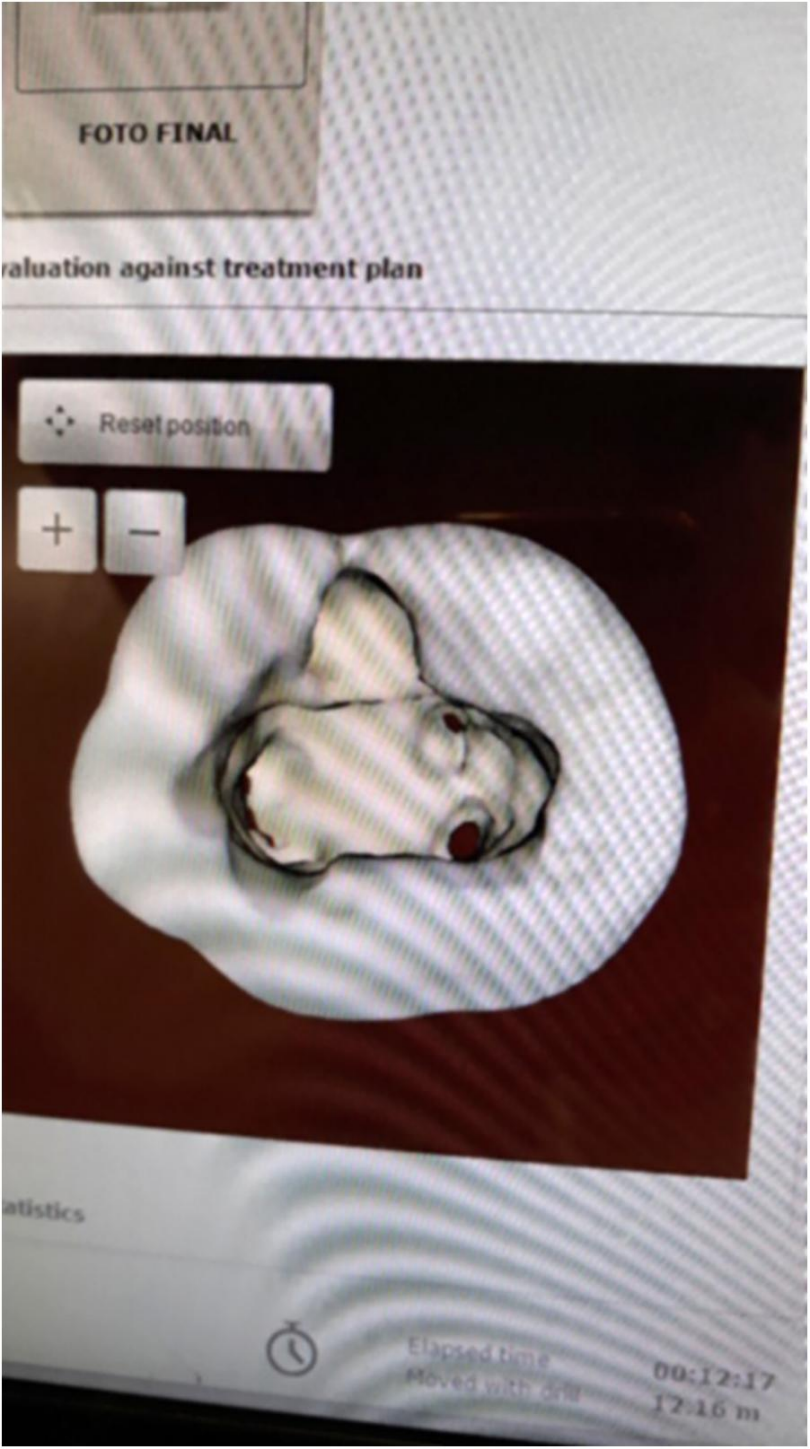
Pulpotomy training in the Simodont® dental trainer. Simulator monitor displaying the pulpotomy exercise.

Students in the experimental group received instructions to perform pulpotomy through the HVR Simodont® Trainer (Nissin Dental Products Europe B.V., Nieuw-Vennep, Netherlands) equipped with software v4.22. The aims of this exercise were to perform a correct pulp chamber opening, considering the extension of the supposed caries lesion and the depth of the pulp chamber, to learn how to shape the access to the pulp chamber with the correct use of endo reamer bur, the correct removal of the dental pulp tissue, and to know the location of pulp horns and the entrance of the root canals. To achieve these objectives, students performed a pulpotomy exercise using the simulator, which required selecting the appropriate burs, removing the carious tissue, accessing the pulp chamber, and conservatively preparing the access cavity with parallel walls (Figure 2).

All students were required to achieve at least 75% of the target, with a 0.3 mm (5%) maximum leeway in both bottom and sides areas, using direct vision, under continuous faculty supervision and guidance. Each student was given a total of 15 min to complete the training session.

To evaluate students' satisfaction and perceived usefulness of HVRS, a questionnaire was administered (3). To ensure equal exposure to the training modality and allow for comparative perception analysis, the control group completed the HVRS session retrospectively, after the main data collection phase.

#### 2.3.2 Conventional training

**Figure 3:**
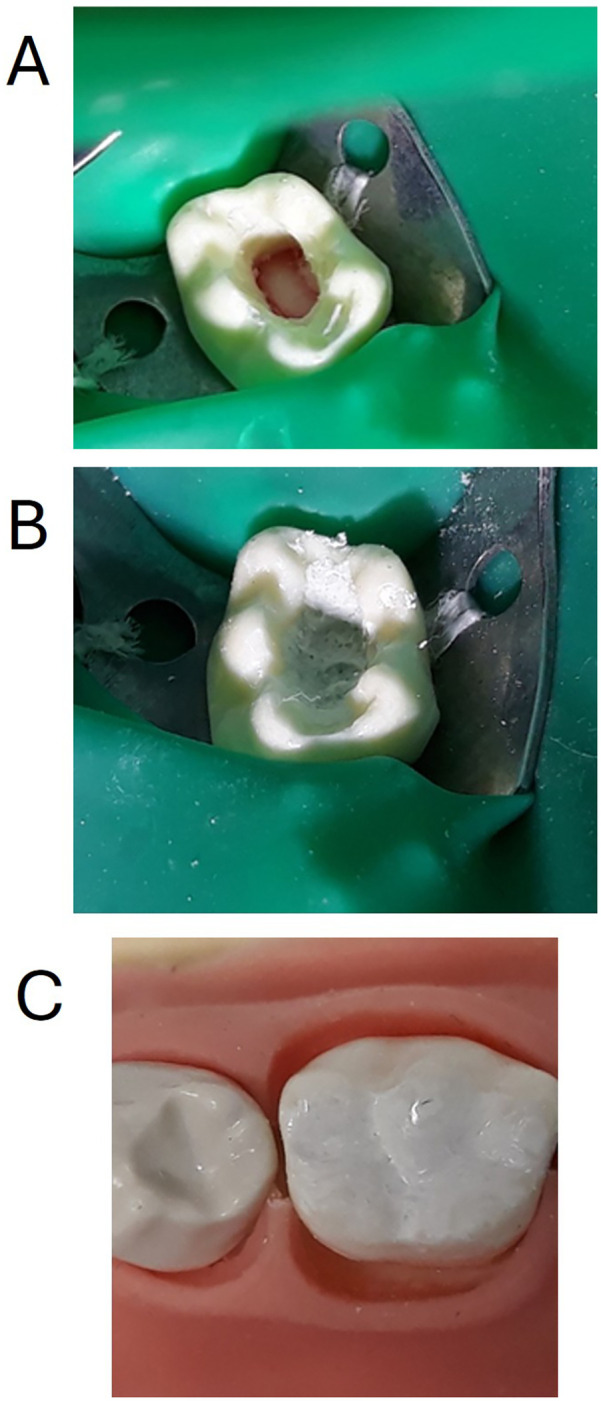
**(A)** An 8.5 primary molar resin tooth (ZPUW 8.5 molar with artificial pulp, frasaco®) integrated into the AK-6/2 child model typodont (Frasaco®) and mounted on Frasaco® mannequins. Opening of the pulp chamber, cavity walls preparation and removal of the pulp chamber tissue has been performed. **(B)** Application of bioactive material: mineral trioxide aggregate (MTA ref. 821, Angelus®) used as a vital pulpotomy material. **(C)** Tooth has been restored with reinforced zinc oxide and eugenol cement (IRM, Denstply®, ref. 60661510).

All participating students were required to perform pulpotomy procedures on 8.5 primary molar resin tooth (ZPUW 8.5 molar with artificial pulp) integrated into the AK-6/2 child model typodont mounted on Frasaco® mannequins). The preclinical practical sessions followed standardized clinical protocols (13), replicating the essential clinical steps of a pulpotomy procedure in pediatric patients, and were carried out as follows. The mannequin was placed in supine position, the student was seated between 9 and 12 o'clock ergonomic operator position; rubber dam total isolation (indirect technique) of tooth 8.5 was carried out using clamp no26N (ref. 50057728, Yvory®) (Figure 3).

Access to the pulp chamber as conservatively as possible was then performed using high-speed air-driven handpiece turbine model (NSK S-Max M®, NSK Dental, Spain) with a round-end tapered, coarse grit (green) diamond bur (Komet®, Dusseldorf, Germany) first, and once the mesiovestibular pulp horn was reached, an endo reamer bur with safe end (Komet®, Dusseldorf, Germany) was used to eliminate completely the roof of the pulp chamber and provide a conservative opening with parallel, smooth, homogeneous walls. Then, all the remaining pulp chamber tissue like material was removed by means of a spoon excavator 1.5 mm (LM-ErgoSense®, LM-DentalTM, Parainen, Finland), physiological saline was applied to the pulp stumps with a sterilized cotton pellet for 2–5 min with slight pressure for hemostasis purposes (Figure 3).

Once supposed hemostasis was achieved, the application of a bioactive material was done using mineral trioxide aggregate (MTA ref. 821, Angelus®) (Figure 3). Finally, the tooth was restored with zinc oxide and eugenol cement (IRM, Denstply®, ref. 60661510) (Figure 3).

### 2.4 Outcomes

**Primary outcomes:** Students' performance in the simulated pulpotomy was assessed with a structured rubric including four predefined technical criteria: (i) Cavity shape: evaluation of the external morphology and outline of the access cavity, in accordance with pediatric endodontic standards. (ii) Conservative opening: evaluation of whether the cavity design preserved the maximum amount of healthy tooth structure while still providing adequate access to the pulp chamber. (iii) Roof removal: verification of complete removal of the pulp chamber roof to ensure proper visibility and access to the pulp chamber. (iv) Pulp chamber configuration: assessment of the internal contour and depth of the pulp chamber, including proper identification and exposure of the canal entrances.

Each criterion was independently scored on a three-point performance scale: Excellent Work (score = 3), indicating optimal execution of the criterion; Heading in the Right Direction (score = 2), reflecting acceptable but improvable performance; Needs Work (score = 1), denoting insufficient or incorrect execution requiring significant improvement (Table 1). The total performance score (maximum 12 points) was calculated by summing the individual scores from the four criteria.

**Table 1:** Competency-based rubric for the pulpotomy assessment.

Criterion	Score 3	Score 2	Score 1
Cavity Shape	Triangular	Partially triangular	Non-triangular
Conservative Opening	Preserves wall and margin integrity	Minimal superficial damage	Significant compromise
Roof Removal	Complete (80%–100%)	Partial (50%–79%)	Incomplete (0%–49%)
Pulp Chamber Configuration	Full visualization of all canal entries	Partial visualization (2 canals)	Incomplete (0–1 canals)

**Secondary outcomes:** Upon completion of the practical sessions, all participants completed a questionnaire from Philip et al., previously used in their preclinical pediatric study to assess students' perceptions of HVRS as a complementary educational tool (3). The instrument included closed-ended items rated on a 5-point Likert scale and four open-ended questions aimed at exploring qualitative aspects of the students' experience (Supplementary Material).

The questionnaire was administered anonymously and on a voluntary basis to both the control and experimental groups one week following the HVRS intervention, in order to assess participants' perceptions while the experience remained temporally proximate and cognitively salient.

**Calibration of examiners:** Prior to the study, two evaluators underwent a calibration process supervised by an external clinical trainer. For this purpose, 10 standardized pulpotomy procedures on mannequin-based models were independently assessed by both examiners at two different time points. The resulting scores were compared within and between examiners, and intra- and inter-examiner agreements were calculated using Cohen's Kappa coefficient to ensure reliability of the rubric scoring system.

### 2.5 Randomization

Participants were randomly assigned to either the control or experimental group using a stratified block randomization method based on prior academic performance to ensure baseline homogeneity. The random allocation sequence was generated electronically by the principal investigators. Allocation concealment was maintained using sequentially numbered, sealed, opaque envelopes, which were prepared in advance. At the time of group assignment, envelopes were opened by a secondary researcher who was not involved in training delivery or outcome assessment. This procedure ensured that allocation remained concealed until the point of assignment.

### 2.6 Blinding

This was a single-blind trial. Due to the nature of the interventions, students could not be blinded to their assigned training modality. However, the evaluators responsible for assessing rubric scores were blinded to group allocation throughout the evaluation process. In addition, the principal investigators who performed the statistical analyses remained blinded to group assignments during data analysis in order to minimize potential bias.

### 2.7 Sample size

The calculation targeted the primary outcome (total pulpotomy performance score, 0–12). A standard deviation (SD ≈ 1.3) was assumed, consistent with variability reported in preclinical HVRS pulpotomy work (3) and comparable preclinical endodontic training with VR haptics (9). A difference of 0.67 units between groups was considered relevant, equivalent to approximately 0.5 SD (Cohen's d ≈ 0.5), which represents a moderate effect size widely adopted in educational and clinical research (14–17). With alpha = 0.05 and power = 90%, G*Power 3.1.9.7 (*t-tests: Means—Difference between two independent means, two groups*) indicated a required sample size of 80 students per group (160 total). Allowing for a 10% dropout, the initial population of 231 students was sufficient.

### 2.8 Statistical methods

The student was considered as the unit of analysis. Descriptive statistics (means and standard deviations) were calculated for each of the evaluated variables.

To test the normality of the distribution of quantitative variables, the Shapiro–Wilk test was applied (*p* > 0.05 for all variables, confirming normal distribution). Levene's test was used to assess the homogeneity of variances (homoscedasticity). Comparisons between the control and experimental groups were performed using the independent samples t-test, provided the assumptions of normality and equal variance were met. Specifically, mean scores for each rubric criterion (cavity shape, conservative opening, roof removal, pulp chamber configuration) and the total performance score were compared between groups.

All data were analyzed using the Statistical Package for the Social Sciences (SPSS), version 29 (IBM Corp., Armonk, NY, USA). The level of statistical significance was set at *p* < 0.05 for all analyses.

## 3 Results

### 3.1 Participant flow

A total of 231 students were initially considered (reference population). Of these, 43 were excluded for not meeting the inclusion criteria (27 had not completed the required prior training and 16 had not passed the Pediatric Dentistry I course). The eligible population therefore comprised 188 students. Among them, 15 were excluded (13 declined participation and 2 were unable to commit to follow-up), resulting in a final study population of 173 students. Participants were randomized into the control group (*n* = 81) and the HVRS group (*n* = 92). All students received the assigned intervention, completed the follow-up, and were included in the final analysis (Figure 1).

### 3.2 Baseline characteristics

All participants belonged to the same academic cohort, with a homogeneous age range and training background. Demographic variables such as age and sex were not collected. Baseline comparability between groups was addressed through stratified block randomization based on prior academic performance.

### 3.3 Primary outcomes

The rubric scores are presented in Table 2. No statistically significant differences were observed in cavity shape and conservative opening scores. However, the experimental group performed significantly better in roof removal (*p* < 0.01), while the control group showed slightly higher scores in pulp chamber configuration (*p* > 0.05). The total score was marginally higher in the experimental group than in the control group, although this difference was not statistically significant (*p* > 0.05).

**Table 2:** Students’ performance score for each evaluation criterion of the control and simodont group.

Criterion	Control group (*n* = 81)	Test group (*n* = 92)	*p*-value
Cavity Shape	1.65 ± 0.73 (1.49–1.82) Median = 2 (1–3)	1.65 ± 0.70 (1.52–1.80) Median = 2 (1–3)	0.984
Conservative Opening	2.17 ± 0.77 (2.00–2.34) Median = 2 (1–3)	2.13 ± 0.81 (1.96–2.30) Median = 2 (1–3)	0.727
Roof Removal	2.42 ± 0.54 (2.30–2.54); Median = 2 (1–3)	2.70 ± 0.51* (2.59–2.80) Median = 3 (1–3)	<0.001
Pulp Chamber Configuration	2.96 ± 0.25 (2.91–3.02) Median = 3 (1–3)	2.84 ± 0.48* (2.74–2.94) Median = 3 (1–3)	0.033
Total score	9.21 ± 1.40 (8.90–9.52) Median = 9 (6–12)	9.32 ± 1.32 (9.04–9.59) Median = 9 (6–12)	0.348

Values are presented as mean ± SD (95% confidence interval); medians and ranges (minimum–maximum) are also reported. *p < 0.05.

Intra-examiner agreements were substantial for both examiner A (κ = 0.840) and examiner B (κ = 0.868), while inter-examiner agreements showed moderate consistency (κ = 0.612).

### 3.4 Secondary outcomes

A total of 135 students completed the post-intervention questionnaire evaluating their experience with the Simodont® simulator (Supplementary Material). Most participants evaluated HVRS positively: 68.2% agreed that the simulator helped them better understand the pulpotomy procedure, 74.3% rated the visual representation as realistic, and 70.6% reported realistic tactile feedback. However, only 47.1% agreed that the deroofing experience resembled plastic teeth. Regarding skill development, 57.3% reported improvement in fine motor skills, and 60.3% indicated increased confidence. Responses were divided on whether HVRS could replace plastic-tooth training (41.5% agreed vs. 40.8% disagreed). Qualitative feedback highlighted advantages such as repeatability, reduced anxiety, and realism of textures, while limitations included partial tactile realism and instrument glitches. Finally, a strong preference emerged for completing simulator training prior to typodont-based practice, with most students reporting that early exposure to HVRS helped reduce anxiety and improve procedural comprehension (Supplementary Material).

### 3.5 Harms

No harms or adverse events were observed in either the control or HVRS group during the course of the study.

## 4 Discussion

This study investigated the impact of HVRS on the preclinical training of pulpotomy in primary molars. The findings suggest that while overall performance between groups was comparable, students who underwent HVRS demonstrated significantly better outcomes in the removal of the pulp chamber roof. These results support the anticipated positive impact of HVRS on specific technical skills and student satisfaction, leading to a partial acceptance of the initial hypothesis. This aligns with prior evidence indicating that HVRS can enhance fine psychomotor skills in operative dentistry, particularly in tasks requiring visual-spatial precision and controlled instrumentation (3, 10, 18).

The enhancement observed in roof removal may reflect the capacity of HVRS to support procedural planning and mental visualization. By allowing deliberate practice in a stress-free setting, the simulator may function as a cognitive scaffold that aids in the internalization of stepwise techniques, a mechanism previously proposed in simulation-based dental education (2, 7). The positive evaluation of simulator realism by the majorityof students further reinforces its pedagogical potential. Specifically, 74.3% perceived the visual representation of the teeth, pulp chamber, and instruments as realistic, and 70.6% could distinguish between enamel and dentine textures. These findings are consistent with prior studies highlighting the importance of visual and tactile fidelity in enhancing student engagement and perceptual learning (9). Notably, haptic feedback has been shown to facilitate the development of tactile discrimination, which is fundamental for tissue management in pediatric and operative dentistry (5).

In terms of affective and behavioral outcomes, more than half of the students reported improved confidence and fine motor skills following simulator use. Confidence in procedural performance has been previously associated with lower error rates and more efficient clinical decision-making during the transition from simulation to patient care (19). HVRS may thus contribute not only to technical proficiency but also to the development of professional self-efficacy, an essential component of competency-based curricula. Another important benefit of HVRS comes from the student's detailed performance, precise parameters and instant assessment that the immediate feedback devices offer, which enables the educator to make those necessary changes within the task that a tailored teaching program might require, so that the student can achieve the subject's learning outcome at their own pace, making this tool an inclusive one (4). Despite these benefits, certain aspects of the simulation received more critical feedback. Notably, only 47.1% of students agreed that deroofing the pulp chamber on the simulator felt similar to the procedure on plastic tooth models, while 30.1% disagreed or strongly disagreed. This discrepancy may be attributed to limitations in current haptic technology when replicating complex, multi-layered tissue resistance, especially in endodontic access procedures. Similar limitations have been noted in previous studies, suggesting that while HVRS excels in basic operative and cavity design training, it may require further refinement for procedures involving fine tissue manipulation and internal anatomy navigation (1, 18). The potential risk of cybersickness should also be considered. Students may experience dizziness and headaches if the simulator is not properly calibrated and adapted for every user, a potential harm that is well documented in other fields (20) but is scarce at the moment in dental education, which opens a new research area in this field.

A particularly relevant result is the polarized opinion on whether HVRS should replace conventional preclinical training. Only 41.5% supported its replacement, while a nearly equal proportion disagreed. These findings underscore the perception of HVRS as a complementary, rather than substitutive, pedagogical tool—an approach supported by recent literature advocating for blended simulation models (6, 21). Open-ended responses from students supported this view, emphasizing the simulator's value for repeated practice, immediate feedback, and early-stage learning, but also highlighting its lack of clinical variability, soft tissue representation, and patient management elements.

From a curricular perspective, the data suggests several practical implications for integrating HVRS into preclinical training. Early exposure to HVRS was associated with improved student confidence and understanding of procedural steps, supporting its value as an introductory, low-risk training tool. The strong preference for simulator use prior to mannequin-based exercises aligns with scaffolded instructional models, potentially enhancing progressive skill development. Although formative assessment was not formally assessed in this study, students' qualitative responses highlighted the value of immediate feedback, suggesting potential utility in this domain. As reported in previous studies (5), HVRS has the potential to standardize practice opportunities and reduce material waste. However, reported technical limitations highlight the importance of continued software refinement and the need for faculty calibration and pedagogical support during implementation (21).

From an educational perspective, HVRS could be progressively integrated into the regular dental curriculum, initially as a complement to traditional simulation in the preclinical phase and, subsequently, as a preparatory tool for clinical training. Its adoption in routine teaching should be accompanied by cost–effectiveness analyses to inform institutional decision-making and ensure sustainable implementation.

This study has several limitations. First, outcomes related to simulator perceptions were self-reported, introducing the risk of response bias. Second, the study was conducted in a single institution, which limits external validity and, therefore, the generalizability of our findings. In this regard, demographic variables such as age and gender were not collected, since all participants belonged to the same academic cohort with a homogeneous age range and comparable training background. While this homogeneity may reduce confounding, it also restricts external validity, as the lack of demographic, socioeconomic, and cognitive data (e.g., IQ) constrains the generalizability of the findings to broader populations and contexts. Third, while student performance was evaluated using a structured rubric, long-term clinical outcomes and retention of skills were not assessed. Another limitation is the absence of clinical trial registration. Although the research was conducted in a preclinical educational setting without direct patient interventions, retrospective registration would enhance transparency and accountability. Future research should include longitudinal follow-up, objective assessment tools, comparisons across institutions and training contexts, and formal trial registration in line with CONSORT recommendations.

As HVRS technology continues to evolve, further studies should explore improvements in haptic feedback precision, enhanced anatomical realism, and its role in simulating comprehensive clinical environments, including patient interaction and decision-making scenarios. Beyond the scope of the present study, haptic virtual reality simulation could also be applied to other complex areas of dentistry that require highly precise training, such as oral surgery, implantology, and periodontology, among others. Further research is warranted to evaluate its effectiveness and potential benefits in these disciplines. Future studies should explore its objective impact of HVRS on clinical performance, long-term skill retention, and its role in competency-based assessments. Continued technological development and pedagogical refinement will be essential to maximize the educational potential of haptic simulation in dentistry.

## 5 Conclusion

Within the limitations of this preliminary study, the integration of HVRS into preclinical training for pediatric pulpotomy demonstrated favourable outcomes particularly in enhancing students' procedural understanding, tactile awareness, and psychomotor development. HVRS was also positively perceived by students as a complementary tool in pediatric preclinical training.

## Data Availability

The raw data supporting the conclusions of this article will be made available by the authors, without undue reservation.
