# Supplementary material for: Can haptic reinforced VR simulation transform preclinical pulpotomy training? Insights into skill acquisition, student perceptions, and educational impact: randomized controlled trial
Source: Front Oral Health. 2025 Sep 24;6:1677056. doi: 10.3389/froh.2025.1677056 (PMC12504311; doi:10.3389/froh.2025.1677056)
Supplement: Supplementary file 1 [file Datasheet1.pdf]

Supplementary Material (S).

S1. Protocol

Diagram of the study protocol.

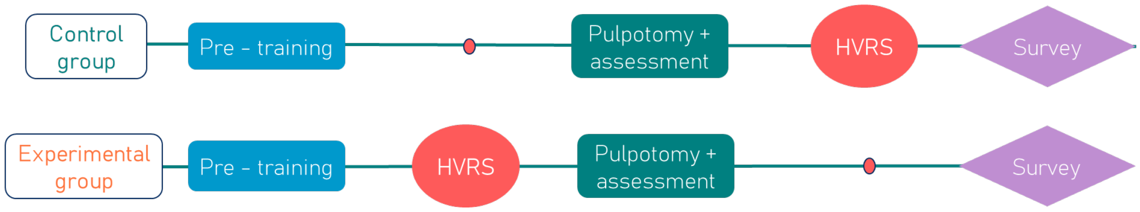

S2. Student perception questionnaire

Student perception questionnaire on pre-clinical haptic virtual reality simulation (HVRS) training. The table lists the closed-ended items rated on a 5-point Likert scale (1 = completely disagree; 2 = disagree; 3 = neutral; 4 = agree; 5 = completely agree) and the open-ended questions used to collect qualitative feedback.

**Closed-ended questions**<br>1. Pulpotomy demonstration on the simulator allowed me to clearly comprehend the tasks expected from me.<br>2. Images of the teeth, pulp chamber, and instruments displayed on the simulator monitor looked realistic.<br>3. I could differentiate between the texture and hardness of enamel and dentine in the simulator device.<br>4. Tactile force feedback given by the simulator felt realistic.<br>5. Deroofing the pulp chamber on the simulator device felt similar to that on plastic teeth mounted on mannequins.<br>6. Training on the simulator device improved my fine motor dental skills.<br>7. Training on the simulator device improved my confidence in performing the pulpotomy procedure.<br>8. The simulator can replace conventional pre-clinical training on plastic teeth for the pulpotomy procedure.
**Open-ended questions**<br>9. In your opinion what is the main benefit of simulator training?<br>10. In your opinion what is the main limitation of simulator training?<br>11. How could pre-clinical training for pulpotomy in primary teeth be improved in the future?<br>12. Would you prefer to have simulator training for primary molar pulpotomy before or after training on plastic tooth models?

S3. Rubric scores

Anonymized student performance scores for the pulpotomy assessment.

code	E/C	Forma	Enfoque	Techo	Cámara	Total
1	2	2	3	2	3	10
2	2	1	2	3	3	9
3	2	1	1	3	3	8
4	2	2	1	3	3	9
5	2	2	3	1	3	9
6	2	2	1	3	3	9
7	2	2	2	2	3	9
8	2	2	1	3	3	9
9	2	1	1	3	3	8
10	2	2	2	3	3	10
11	2	3	2	3	3	11
12	2	3	3	3	3	12
13	2	2	3	3	3	11
14	2	3	2	3	3	11
15	2	2	2	3	3	10
16	2	2	2	3	3	10
17	2	2	1	3	3	9
18	2	1	1	2	3	7
19	2	1	1	2	3	7
20	2	2	3	1	1	7
21	2	1	1	3	3	8
22	2	1	2	2	3	8
23	2	2	3	3	3	11
24	1	2	3	3	3	11
25	1	1	2	2	3	8
26	1					
27	1	3	1	2	3	9
28	1	3	2	2	3	10
29	1	2	3	2	3	10
30	1	2	3	3	3	11
31	1	2	2	2	3	9
32	1	3	1	3	3	10
33	2	1	2	3	3	9
34	2	1	2	3	3	9
35	2	1	1	3	3	8
36	2					
37	2	1	1	3	3	8
38	2	1	2	3	3	9
39	2	2	2	3	3	10
40	2	2	2	3	3	10
41	2	2	3	3	3	11
42	2	1	3	3	3	10
43	1	3	3	3	3	12

44	1	1	2	3	3	9
45	1	1	1	3	3	8
46	1	2	2	2	3	9
47	1	3	2	3	3	11
48	1	1	3	2	3	9
49	1	1	2	3	3	9
50	1	2	1	3	3	9
51	1	2	2	2	3	9
52	2	2	1	3	3	9
53	2	1	1	2	3	7
54	2	2	2	3	3	10
55	2	1	1	3	3	8
56	2					
57	2	3	3	2	1	9
58	2	3	2	2	3	10
59	2	1	1	3	3	8
60	2	2	3	3	3	11
61	2	1	1	2	3	7
62	1					
63	1	2	2	2	1	7
64	1	3	3	2	3	11
65	1	3	3	3	3	12
66	1					
67	1	2	2	3	3	10
68	1	1	1	2	3	7
69	1	2	3	3	3	11
70	1	2	1	3	3	9
71	1	2	3	2	3	10
72	1	2	3	2	3	10
73	2	2	3	3	3	11
74	2	1	2	3	3	9
75	2	1	3	3	3	10
76	2	1	2	3	3	9
77	2	1	3	2	3	9
78	2	1	3	3	3	10
79						
80	2	1	2	3	3	9
81	2	1	1	2	3	7
82	2	2	2	3	2	9
83	2	1	2	3	3	9
84	2	1	2	3	1	7
85	2	3	2	3	3	11
86	2	2	1	2	3	8
87	2	1	3	3	2	9
88	2	2	2	3	3	10
89	1	2	1	3	3	9
90	1	1	2	2	3	8
91	1	2	3	2	3	10

92	1	1	2	2	3	8
93	1	1	3	1	3	8
94	1	1	3	2	3	9
95	1	1	3	1	3	8
96	1	1	2	2	3	8
97	1	2	2	3	3	10
98	1	1	3	2	3	9
99	1	1	1	2	3	7
100	1	2	3	2	3	10
101	1	2	3	2	3	10
102	1	1	1	3	3	8
103	1	2	3	3	3	11
104	1	1	3	2	3	9
105	1	1	2	2	3	8
106	1	1	2	2	3	8
107	1	1	2	2	3	8
108	2	1	3	3	3	10
109						
110	2	2	3	2	3	10
111	2	1	3	3	3	10
112	2	2	3	2	3	10
113	2	1	3	2	2	8
114	2	2	3	3	3	11
115	2	1	1	3	2	7
116	2	2	3	3	3	11
117	2	2	3	3	3	11
118	2	1	3	3	3	10
119						
120	2	2	3	3	3	11
121	2	1	1	3	3	8
122	2	3	3	2	3	11
123	2	3	1	3	3	10
124	2	1	3	2	3	9
125	2	1	2	3	3	9
126	2	1	3	3	3	10
127	2	2	3	2	3	10
128	1	2	2	2	3	9
129	1	2	2	3	3	10
130	1	3	3	3	3	12
131	1	1	1	3	3	8
132	1	2	3	3	3	11
133	1	2	3	3	3	11
134	1	2	2	2	3	9
135	1	2	3	3	3	11
136	1	3	3	3	3	12
137	1	1	1	3	3	8
138	1	1	1	3	3	8

139	1	3	3	3	3	12
140	1	1	2	2	3	8
141	1	3	3	3	3	12
142						
143	1	3	3	3	3	12
144	1	2	2	2	3	9
145	1	2	2	2	3	9
146	1	2	2	2	3	9
147	1	1	3	2	3	9
148	2	1	1	3	3	8
149	2	2	3	3	2	10
150	2	2	2	3	3	10
151	2	2	2	2	3	9
152	2	3	3	3	3	12
153	2	2	2	3	3	10
154	2	1	3	3	3	10
155	2	1	1	2	2	6
156	2					
157	2	1	2	2	2	7
158	2	2	1	3	3	9
159	2	3	3	3	3	12
160	2	1	3	3	3	10
161	2	3	3	2	3	11
162	2	2	2	3	1	8
163	2	1	3	2	3	9
164	2	3	1	3	3	10
165	2	1	3	2	3	9
166	2	1	2	3	3	9
167	2					
168	1	1	3	2	3	9
169	1					0
170	1	1	1	2	2	6
171	1					
172	1	1	2	3	3	9
173	1	1	3	2	3	9
174	1	1	2	2	3	8
175	1					
176	1	1	3	2	3	9
177	1	1	3	3	3	10
178	1	1	2	2	3	8
179	1	2	1	2	3	8
180	1	1	2	2	3	8
181	1	1	1	2	3	7
182	1	1	2	3	3	9
183	1	1	1	2	3	7
184	1	1	1	3	3	8
185	1	1	2	3	3	9

186	1	1	1	3	3	8
187	1	1	2	3	3	9

S4. Questionnaire responses

Anonymized dataset of student responses to the perception questionnaire.

ID	He sido informado/a sobre los beneficios que podría suponer la participación en el Proyecto "Rúbricas para la evaluación de competencias en las prácticas de laboratorio de Ciencias Básicas del Gra...	¿Eres repetidor de esta asignatura? / Are you a repeat student in this course ?	Que edad tienes ? / How old are you?	¿Cual es tu sexo ? / What is your sex?	¿Cuál es tu país de nacimiento? / What is your country of birth?	La demostración de la pulpotomía en el simulador me permitió comprender claramente los pasos que se esperaban de mí. / Pulpotomy demonstration on the simulator allowed me to clearly comprehend the...	Las imágenes del diente, la cámara pulpar y los instrumentos mostrados en el simulador parecían reales. / Images of the teeth, pulp chamber, and instruments displayed on the simulator monitor look...	Se podía diferenciar entre la textura y dureza del esmalte y la dentina en el simulador. / I could differentiate between the texture and hardness of enamel and dentine in the simulator device.	La fuerza táctil que se hace con el simulador parecía realista. / Tactile force feedback given by the simulator felt realistic.	La apertura de la cámara pulpar en el simulador se sentía similar a los dientes utilizados en el tipodonto. / Deroofing the pulp chamber on the simulator-device felt similar to that on plastic teet...	El uso del simulador ha mejorado mis habilidades en en este tratamiento dental / Training on the simulator device improved my fine motor dental skills.	El entrenamiento con el simulador ha mejorado mi confianza en la realización de la pulpotomía dental. / Training on the simulator device improved my confidence in performing the pulpotomy procedure.	El simulador puede sustituir perfectamente la práctica pre clínica de la pulpotomía sobre dientes de resina. / The simulator can replace conventional pre-clinical training on plastic teeth for the...	En tu opinón, ¿cuál es el mayor beneficio de la práctica sobre el simulador?/ In your opinion what is the main benefit of simulator training?	En tu opinión, ¿Cuál es la principal limitación en la práctica con el simulador?/ In your opinion what is the main limitation simulator of training?	¿Cómo podría mejorarse en el futuro la práctica clínica de la pulpotomía en dientes deciduos? / How could preclinical training for pulpotomy in primary teeth be improved in the future?	¿Preferiría tener la practice de pulpotomía con el simulador antes o después de la práctica con dientes de resina?/ Would you prefer to have simulator training for primary molar pulpotomy before ...

2	Si / Yes	Sí / Yes	22	Hombre / Male	USA	de acuerdo / agree	de acuerdo / agree	de acuerdo / agree	de acuerdo / agree	de acuerdo / agree	de acuerdo / agree	de acuerdo / agree	de acuerdo / agree	como la realidad	neutro	neutro	antes
3	Si / Yes	No	22	Mujer / Female	Francia	neutral	neutral	neutral	neutral	en desacuerdo / disagree	neutral	neutral	en desacuerdo / disagree	Practicar y repetir	La textura	Realizar más prácticas	Antes
4	Si / Yes	No	19	Mujer / Female	France	de acuerdo / agree	completamente de acuerdo / completely agree	completamente de acuerdo / completely agree	de acuerdo / agree	en desacuerdo / disagree	neutral	neutral	de acuerdo / agree	sentir la textura real de un diente	no tener el número de instrumentos ilimitados	mostrar paso a paso para que los alumnos visualizan mejor	Antes
5	Si / Yes	No	21	Mujer / Female	Francia	de acuerdo / agree	de acuerdo / agree	completamente de acuerdo / completely agree	completamente de acuerdo / completely agree	de acuerdo / agree	de acuerdo / agree	de acuerdo / agree	de acuerdo / agree	Tener una visión de la clínica	No es real real	No se	Si
7	Si / Yes	No	22	Mujer / Female	France	de acuerdo / agree	completamente de acuerdo / completely agree	completamente de acuerdo / completely agree	completamente de acuerdo / completely agree	de acuerdo / agree	completamente de acuerdo / completely agree	completamente de acuerdo / completely agree	en desacuerdo / disagree	Es que es un buen entrenamiento y parece real	No estamos en conditiones clinicas y todo esta diferente cuando estamos con un paciente real	Hacerlo mas veces en el fantoma	Antes
8	Si / Yes	No	22	Mujer / Female	21/03/2003	neutral	de acuerdo / agree	de acuerdo / agree	completamente de acuerdo / completely agree	neutral	neutral	neutral	en desacuerdo / disagree	podemos sentir el tacto de una real diente	no es muy real porque podemos aproximarnos	no se	antes

															mucho del diente		
9	Si / Yes	No	23	Mujer / Female	España	completamente de acuerdo / completely agree	de acuerdo / agree	completamente de acuerdo / completely agree	de acuerdo / agree	en desacuerdo / disagree	de acuerdo / agree	de acuerdo / agree	completamente de acuerdo / completely agree	se siente muy real	el poco tiempo que tenemos		antes
10	Si / Yes	No	22	Mujer / Female	Francia	de acuerdo / agree	completamente de acuerdo / completely agree	completamente de acuerdo / completely agree	completamente de acuerdo / completely agree	de acuerdo / agree	de acuerdo / agree	de acuerdo / agree	de acuerdo / agree	para entender como hacer en la realidad	la ergonomia et la posicion de la boca	conozco las diferentes zonas	antes
11	Si / Yes	No	21	Mujer / Female	Francia	neutral	de acuerdo / agree	de acuerdo / agree	de acuerdo / agree	de acuerdo / agree	neutral	neutral	completamente en desacuerdo / completely disagree	Simular la realidad	Los instrumentos estan muy grandes y enmascaran la vision	Practicar mas	Antes
12	Si / Yes	Sí / Yes	28	Mujer / Female	Francia	completamente de acuerdo / completely agree	completamente de acuerdo / completely agree	completamente de acuerdo / completely agree	completamente de acuerdo / completely agree	completamente de acuerdo / completely agree	completamente de acuerdo / completely agree	completamente de acuerdo / completely agree	completamente de acuerdo / completely agree	Poder repetir tantas veces que nos hace falta	Ninguna		Antes
13	Si / Yes	No	22	Mujer / Female	Francia	de acuerdo / agree	de acuerdo / agree	de acuerdo / agree	de acuerdo / agree	en desacuerdo / disagree	completamente de acuerdo / completely agree	completamente de acuerdo / completely agree	en desacuerdo / disagree	Está más realista que en el tipodont	No hay la piel, músculos y todo alrededor de los maxilares	Hacerlo más de una vez	Antes
15	Si / Yes	Sí / Yes	23	Hombre / Male	France	de acuerdo / agree	de acuerdo / agree	en desacuerd	de acuerdo / agree	de acuerdo / agree	en desacuerd	neutral	completamente en desacuerd	Podemos hacer	A veces no es realista	Neutral	Antes

								o / disagree			o / disagree		o / completely disagree	la práctica tantas veces como queremos			
16	Si / Yes	No	20	Hombre / Male	Francia	de acuerdo / agree	neutral	de acuerdo / agree	de acuerdo / agree	neutral	en desacuerdo / disagree	en desacuerdo / disagree	neutral	Praticar	El tacto	No se	Antes
17	Si / Yes	No	21	Hombre / Male	Francia	de acuerdo / agree	de acuerdo / agree	de acuerdo / agree	de acuerdo / agree	de acuerdo / agree	neutral	neutral	en desacuerdo / disagree	Repetit varias veces	No es totalmente real	Mas tiempo para practicar	Mejor antes de la practica
18	Si / Yes	No	21	Mujer / Female	Francia	de acuerdo / agree	de acuerdo / agree	completamente de acuerdo / completely agree	neutral	neutral	en desacuerdo / disagree	neutral	de acuerdo / agree	Entrenamiento		No se	Antes
19	Si / Yes	No	24	Mujer / Female	Francia	neutral	neutral	de acuerdo / agree	de acuerdo / agree	neutral	en desacuerdo / disagree	en desacuerdo / disagree	completamente en desacuerdo / completely disagree	Tener las bases	Esta bien pero 1 no es suficiente	Mas explicación	Antes
20	Si / Yes	No	23	Mujer / Female	france	completamente de acuerdo / completely agree	completamente de acuerdo / completely agree	completamente de acuerdo / completely agree	de acuerdo / agree	de acuerdo / agree	neutral	de acuerdo / agree	completamente en desacuerdo / completely disagree	podemos empezar de nuevo	alguna veces llegamos a pulpa sin tocar nada casi	no se	antes
21	Si / Yes	No	23	Mujer / Female	France	completamente de acuerdo / completely agree	de acuerdo / agree	completamente de acuerdo / completely agree	completamente de acuerdo / completely agree	completamente de acuerdo / completely agree	completamente de acuerdo / completely agree	completamente de acuerdo / completely agree	completamente de acuerdo / completely agree	De sentir las diferentes texturas	La vista	nada	antes
22	Si / Yes	Sí / Yes	23	Mujer / Female	España	de acuerdo / agree	de acuerdo / agree	de acuerdo / agree	completamente de acuerdo /	de acuerdo / agree	completamente de acuerdo /	de acuerdo / agree	de acuerdo / agree	Mejorar mis habilidades.	Ninguna	No sé	Antes

									completely agree		completely agree						
23	Si / Yes	No	22	Hombre / Male	France	de acuerdo / agree	completamente de acuerdo / completely agree	neutral	de acuerdo / agree	en desacuerdo / disagree	de acuerdo / agree	de acuerdo / agree	en desacuerdo / disagree	Entender lo que es una pulpotomia	Texturas	Empezar por el simodont y hacer despues la practica del tipodont	Antes
24	Si / Yes	Sí / Yes	23	Mujer / Female	Francia	completamente de acuerdo / completely agree	completamente de acuerdo / completely agree	de acuerdo / agree	completamente de acuerdo / completely agree	completamente de acuerdo / completely agree	completamente de acuerdo / completely agree	completamente de acuerdo / completely agree	completamente de acuerdo / completely agree	De ver cómo hacer antes de hacerlo en el fantoma	Que no es la textura real		Si está bien como eso
25	Si / Yes	No	20	Hombre / Male	España	completamente de acuerdo / completely agree	completamente de acuerdo / completely agree	de acuerdo / agree	de acuerdo / agree	neutral	neutral	de acuerdo / agree	completamente en desacuerdo / completely disagree	Nos deja practicar ilimitadas veces con una realidad relativa.	El programa tiene sus fallos aunque es didáctico.	Está bien	Antes
26	Si / Yes	Sí / Yes	24	Hombre / Male	Francia	de acuerdo / agree	de acuerdo / agree	completamente de acuerdo / completely agree	completamente de acuerdo / completely agree	de acuerdo / agree	de acuerdo / agree	de acuerdo / agree	neutral	Puedo hacer error sin consecuencia	No es real	No se	Antes
27	Si / Yes	Sí / Yes	25	Mujer / Female	Italia	de acuerdo / agree	de acuerdo / agree	de acuerdo / agree	de acuerdo / agree	de acuerdo / agree	de acuerdo / agree	neutral	en desacuerdo / disagree	Que podemos	Al principio no entiendo bien		Prefiero antes

														ver antes como se hace la práctica	cuanta fuerza utilizar		
29	Si / Yes	No	22 anos	Hombre / Male	Francia	de acuerdo / agree	de acuerdo / agree	de acuerdo / agree	de acuerdo / agree	neutral	neutral	de acuerdo / agree	de acuerdo / agree	La diferencia entre esmalte y dentina	La realidad de la boca	No se	Sobre una diente de resina
31	Si / Yes	Sí / Yes	23	Hombre / Male	Francia	de acuerdo / agree	de acuerdo / agree	en desacuerdo / disagree	de acuerdo / agree	de acuerdo / agree	neutral	neutral	en desacuerdo / disagree	Cambia el aprendisaje	No es real	En dientes reales	Después
32	Si / Yes	No	22	Mujer / Female	Italia	de acuerdo / agree	completamente de acuerdo / completely agree	completamente de acuerdo / completely agree	completamente de acuerdo / completely agree	completamente de acuerdo / completely agree	de acuerdo / agree	de acuerdo / agree	de acuerdo / agree	Podemos ver mas claramente los diferentes tejidos dentales y entender como usar mejor los instrumentos	Que no es un paciente real y claramente no es como trabajar en un paciente normal	Podemos hacerlo más de una sola vez para practicarlo mejor	Antes para entender mejor y después trabajar con los dientes de resina
33	Si / Yes	No	21	Hombre / Male	France	de acuerdo / agree	de acuerdo / agree	de acuerdo / agree	de acuerdo / agree	de acuerdo / agree	de acuerdo / agree	de acuerdo / agree	de acuerdo / agree	Bueno	No es la realidad no hay paciente	Con verdaderos dientes	Antes
34	Si / Yes	No	21	Hombre / Male	Germany	completamente de acuerdo /	neutral	en desacuerd	en desacuerd	completamente en desacuerd	completamente de acuerdo /	completamente de acuerdo /	en desacuerd	Real feeling	Bugs	The simulator	After

						completely agree		o / disagree	o / disagree	o / completely disagree	completely agree	completely agree	o / disagree			should fix the probe	
35	Si / Yes	No	23	Mujer / Female	The netherlands	de acuerdo / agree	neutral	de acuerdo / agree	de acuerdo / agree	en desacuerdo / disagree	completamente de acuerdo / completely agree	de acuerdo / agree	de acuerdo / agree	Feeling difference between “real” tooth and plastic	Some instruments don’t work like they need to, with excavator you’re going through the tooth the whole time	No idea	Before
36	Si / Yes	No	22	Mujer / Female	Francia	de acuerdo / agree	neutral	completamente de acuerdo / completely agree	completamente de acuerdo / completely agree	completamente en desacuerdo / completely disagree	neutral	de acuerdo / agree	en desacuerdo / disagree	Training	The visual is not realistic	I dont know	Before
37	Si / Yes	No	21	Mujer / Female	Italia	de acuerdo / agree	neutral	de acuerdo / agree	neutral	neutral	de acuerdo / agree	de acuerdo / agree	neutral	La diferencia de los tejidos	El tiempo	Tener más tiempo	Antes
38	Si / Yes	Sí / Yes	27	Hombre / Male	Norwegian	completamente de acuerdo / completely agree	de acuerdo / agree	completamente de acuerdo / completely agree	neutral	neutral	neutral	de acuerdo / agree	completamente en desacuerdo / completely disagree	Gives some understanding of anatomy and procedure	Doesn't feel realistic	More training	Before
39	Si / Yes	No	22	Mujer / Female	France	de acuerdo / agree	de acuerdo / agree	neutral	neutral	de acuerdo / agree	de acuerdo / agree	de acuerdo / agree	en desacuerdo / disagree	Different experience	It’s not real life	Do it in real tooth	Before

40	Si / Yes	No	21	Mujer / Female	Martinique	de acuerdo / agree	de acuerdo / agree	de acuerdo / agree	de acuerdo / agree	en desacuerdo / disagree	de acuerdo / agree	de acuerdo / agree	de acuerdo / agree	Realistic	Good	More explainations from thé professirs	Before
41	Si / Yes	No	25	Mujer / Female	Switzerland	neutral	neutral	de acuerdo / agree	de acuerdo / agree	en desacuerdo / disagree	de acuerdo / agree	neutral	completamente en desacuerdo / completely disagree	Ability to restart the treatment	Sometimes certain instruments have a glitch	Maintain the practice in plastic teeth, but with an improved “pulp”	Before
42	Si / Yes	No	21	Mujer / Female	Italia	completamente de acuerdo / completely agree	en desacuerdo / disagree	completamente de acuerdo / completely agree	completamente de acuerdo / completely agree	completamente de acuerdo / completely agree	completamente de acuerdo / completely agree	completamente de acuerdo / completely agree	completamente en desacuerdo / completely disagree	the fact that it is a good representation of real life	sometimes the instruments don’t work and “pass through” the tooth	i think that it is good as it is	i would prefer to do it after the plastic tooth practice
43	Si / Yes	No	23	Mujer / Female	Senegal	de acuerdo / agree	de acuerdo / agree	neutral	neutral	en desacuerdo / disagree	de acuerdo / agree	de acuerdo / agree	neutral	Improve dexterity	Perforation with certain instruments, limitation on the burs choice		Before
46	Si / Yes	No	21	Hombre / Male	France	de acuerdo / agree	de acuerdo / agree	de acuerdo / agree	de acuerdo / agree	de acuerdo / agree	de acuerdo / agree	de acuerdo / agree	de acuerdo / agree	Retry as much as we want	Lack a bit of real feeling	Practice more on	Before

																phantom	
47	Si / Yes	No	20	Mujer / Female	Italy	de acuerdo / agree	neutral	neutral	de acuerdo / agree	en desacuerdo / disagree	de acuerdo / agree	en desacuerdo / disagree	en desacuerdo / disagree	Better visualization than model	It is not like a real mouth		Before
48	Si / Yes	No	28	Mujer / Female	Germany	de acuerdo / agree	de acuerdo / agree	de acuerdo / agree	neutral	neutral	en desacuerdo / disagree	de acuerdo / agree	neutral	It‘s possible so feel the difference in the composition of the structures, like enamel , dentine and the pulp.	That the instruments and holding them, doesn‘t feel realistic. Which is due to the simulator using the same piece for every instrument.	I think it was fine.	Before was good for me.
49	Si / Yes	No	20	Mujer / Female	France	completamente de acuerdo / completely agree	de acuerdo / agree	en desacuerdo / disagree	neutral	en desacuerdo / disagree	de acuerdo / agree	completamente de acuerdo / completely agree	en desacuerdo / disagree	Have a better view of what expected	For pulpotomy, the different sensation beteween dentine and enamel	Idk	Before
53	Si / Yes	No	27	Hombre / Male	Italia	neutral	de acuerdo / agree	completamente de acuerdo / completely agree	de acuerdo / agree	neutral	de acuerdo / agree	de acuerdo / agree	de acuerdo / agree	Que puedes repetir la simulación	El tiempo	Tener más tiempo para la simulación	Antes

														desde cero			
54	Si / Yes	No	21	Mujer / Female	Italia	de acuerdo / agree	completamente de acuerdo / completely agree	completamente de acuerdo / completely agree	completamente de acuerdo / completely agree	en desacuerdo / disagree	de acuerdo / agree	de acuerdo / agree	de acuerdo / agree	La texture	Los instrumentos		Después
56	Si / Yes	No	22	Mujer / Female	Italia	neutral	de acuerdo / agree	completamente en desacuerdo / completely disagree	de acuerdo / agree	de acuerdo / agree	neutral	neutral	de acuerdo / agree	Para trabajar con la sensibilidad	Visión limitada	Hacerla más veces	Después
60	No	No	23	Mujer / Female	Italia	en desacuerdo / disagree	neutral	de acuerdo / agree	de acuerdo / agree	en desacuerdo / disagree	neutral	neutral	completamente de acuerdo / completely agree	Parece realistico	Que tenemos 5 minutos casa uno	Hacer practicas intera para cada uno en el simulador	Antes
61	Si / Yes	No	20	Mujer / Female	Francia	neutral	de acuerdo / agree	completamente de acuerdo / completely agree	neutral	en desacuerdo / disagree	neutral	de acuerdo / agree	completamente en desacuerdo / completely disagree	repetir muchas veces			antes
62	Si / Yes	Sí / Yes	23	Mujer / Female	Italia	completamente en desacuerdo / completely disagree	completamente en desacuerdo / completely disagree	completamente en desacuerdo / completely disagree	completamente en desacuerdo / completely disagree	completamente en desacuerdo / completely disagree	completamente en desacuerdo / completely disagree	completamente en desacuerdo / completely disagree	completamente en desacuerdo / completely disagree	Entender muy bien las texture del diente y afinar la sensibilidad	Ninguna	En ninguna maniera, está bien así	Antes
64	Si / Yes	No	24	Mujer / Female	Italia	de acuerdo / agree	de acuerdo / agree	de acuerdo / agree	neutral	en desacuerdo / disagree	neutral	neutral	neutral	Se parece a la realidad	Ninguna	.	Antes

65	Si / Yes	No	21	Hombre / Male	España	de acuerdo / agree	de acuerdo / agree	neutral	de acuerdo / agree	de acuerdo / agree	neutral	neutral	de acuerdo / agree	Puede ayudar a mejorar al realizar la práctica	No se me ocurre	Con ayuda del simodont	Antes para practicar
67	Si / Yes	No	21	Hombre / Male	Madagascar	de acuerdo / agree	de acuerdo / agree	de acuerdo / agree	de acuerdo / agree	de acuerdo / agree	de acuerdo / agree	de acuerdo / agree	de acuerdo / agree	Practicar	No es real	No se	No le se
68	Si / Yes	No	23	Hombre / Male	Italia	de acuerdo / agree	de acuerdo / agree	completamente de acuerdo / completely agree	completamente de acuerdo / completely agree	en desacuerdo / disagree	neutral	neutral	en desacuerdo / disagree	La sensibilidad que se puede apreciar en el simodont	Che no se entienden bien las profundidades	Separando la práctica en dos para entender mejor los pasajes	Antes
69	Si / Yes	No	22	Mujer / Female	Francia	de acuerdo / agree	completamente de acuerdo / completely agree	completamente de acuerdo / completely agree	completamente de acuerdo / completely agree	en desacuerdo / disagree	de acuerdo / agree	de acuerdo / agree	de acuerdo / agree	La sensacion real de los dientes	No vemos el diente como si esta en paciente réal podemos hacer zoom pero en la vida no podemos hacerlo	hacer mas practicas	antes
70	Si / Yes	No	20	Mujer / Female	France	neutral	de acuerdo / agree	completamente de acuerdo / completely agree	completamente de acuerdo / completely agree	completamente de acuerdo / completely agree	completamente de acuerdo / completely agree	de acuerdo / agree	de acuerdo / agree	Realismo		Seguir así	Después
71	Si / Yes	No	22	Mujer / Female	Francia	de acuerdo / agree	de acuerdo / agree	completamente de acuerdo /	de acuerdo / agree	de acuerdo / agree	de acuerdo / agree	de acuerdo / agree	de acuerdo / agree	Sensacion réalista	No se	No se	Antes

								completely agree						y permite hacer errores			
72	Si / Yes	No	21	Mujer / Female	Francia	completamente de acuerdo / completely agree	de acuerdo / agree	de acuerdo / agree	completamente de acuerdo / completely agree	de acuerdo / agree	de acuerdo / agree	de acuerdo / agree	neutral	Formación	No hay	La primera vez podemos hacer fuera de la boca	Antes
73	Si / Yes	No	20	Mujer / Female	Francia	completamente de acuerdo / completely agree	completamente de acuerdo / completely agree	de acuerdo / agree	completamente de acuerdo / completely agree	de acuerdo / agree	de acuerdo / agree	de acuerdo / agree	neutral	Saber que no gastamos dientes de resina, y que no es grave si fallamos el tratamiento	No hay	Hacer la pulpotomia primero sobre el diente de resina fuera del tipodonto	Antes
74	Si / Yes	Sí / Yes	21	Mujer / Female	France	completamente de acuerdo / completely agree	completamente de acuerdo / completely agree	completamente de acuerdo / completely agree	completamente de acuerdo / completely agree	completamente de acuerdo / completely agree	completamente de acuerdo / completely agree	completamente de acuerdo / completely agree	completamente de acuerdo / completely agree	Bien	Nada	Bien	Bien
75	Si / Yes	No	20	Mujer / Female	21/12/2004	de acuerdo / agree	de acuerdo / agree	completamente de acuerdo / completely agree	de acuerdo / agree	en desacuerdo / disagree	neutral	neutral	completamente en desacuerdo / completely disagree	ver una situación parecida a la realidad	que no tienes la sensación de la realidad		si, porque así se ve menos real al principio y luego más real
76	Si / Yes	Sí / Yes	21	Mujer / Female	España	de acuerdo / agree	de acuerdo / agree	neutral	neutral	neutral	de acuerdo / agree	de acuerdo / agree	completamente en desacuerd	Que la imagen es más realista	Lo puedes repetir todas las		antes

												o / completely disagree		veces que quieras y no lo haces con tanta precisión		
77	Si / Yes	No	22	Hombre / Male	Italiy	de acuerdo / agree	de acuerdo / agree	de acuerdo / agree	de acuerdo / agree	de acuerdo / agree	de acuerdo / agree	de acuerdo / agree	de acuerdo / agree	Practicing	Too mechanic	Don't know	Before
78	Si / Yes	No	20	Mujer / Female	Francia	de acuerdo / agree	neutral	neutral	de acuerdo / agree	en desacuerdo / disagree	neutral	en desacuerdo / disagree	completamente en desacuerdo / completely disagree	Mimicar una situación real con tejidos reales que no tenemos con los modelos de resina	La manera de coger el material, a veces se va de la pantalla y se tiene que buscar, además se ponían con dientes solos y no dentro de la boca	Hacerlo con verdaderos dientes deciduos que se pueden poner en un modelo	Prefiero tener el simulador antes que la resina
79	Si / Yes	No	28	Mujer / Female	Ecuador	de acuerdo / agree	de acuerdo / agree	de acuerdo / agree	de acuerdo / agree	neutral	neutral	neutral	de acuerdo / agree	Visualizar mejor la pulpa	Poco entrenamiento. La simulación es muy apurada	Hacer una clase dedicada a eso y no hacerlo con otras practicas	Antes

80	Si / Yes	No	22	Mujer / Female	España	de acuerdo / agree	neutral	de acuerdo / agree	de acuerdo / agree	de acuerdo / agree	de acuerdo / agree	de acuerdo / agree	de acuerdo / agree	Conocer la textura de un diente real	Nonguna	-	Antes
81	Si / Yes	No	24	Mujer / Female	Francia	completamente de acuerdo / completely agree	completamente de acuerdo / completely agree	completamente de acuerdo / completely agree	completamente de acuerdo / completely agree	completamente de acuerdo / completely agree	completamente de acuerdo / completely agree	completamente de acuerdo / completely agree	completamente de acuerdo / completely agree	En mi opinión, el mayor beneficio de la práctica sobre el simulador es que puedes aprender y mejorar tus habilidades sin riesgo, antes de trabajar con un paciente real.	En mi opinión, la principal limitación de la práctica con el simulador es que no es exactamente como trabajar con un paciente real. No se sienten las mismas texturas, reacciones o imprevistos, así que la experiencia real siempre es diferente.	Simuladores más realistas	Preferiría tener la práctica con el simulador antes de trabajar con dientes de resina, porque el simulador me ayuda a entender mejor los pasos del procedimiento y a ganar confianza. Luego, con los dientes de resina, puedo aplicar lo aprendido con más precisión.
83	Si / Yes	Sí / Yes	23	Mujer / Female	españa	de acuerdo / agree	neutral	neutral	neutral	neutral	de acuerdo / agree	neutral	en desacuerdo / disagree	se nota ms la resistencia qu en un	no es muy real	siendo un poco más realista	antes

														diente de resina			
84	Si / Yes	No	24	Hombre / Male	Francia		completamente en desacuerdo / completely disagree	completamente en desacuerdo / completely disagree	completamente en desacuerdo / completely disagree	completamente en desacuerdo / completely disagree	completamente en desacuerdo / completely disagree	completamente en desacuerdo / completely disagree	completamente en desacuerdo / completely disagree	Aprender	Realismo	No hacerlo en 10 minutos	Despues
85	Si / Yes	No	21	Hombre / Male	Argentina	de acuerdo / agree	de acuerdo / agree	neutral	completamente de acuerdo / completely agree	completamente de acuerdo / completely agree	de acuerdo / agree	completamente de acuerdo / completely agree	en desacuerdo / disagree	Poder realizar una pulpotomia las veces que sea necesario en la misma sesión	La visión y manipulación del diente	Tener más practicas sobre la misma	Antes
86	Si / Yes	No	23	Mujer / Female	Francia	en desacuerdo / disagree	neutral	en desacuerdo / disagree	neutral	completamente en desacuerdo / completely disagree	en desacuerdo / disagree	completamente en desacuerdo / completely disagree	en desacuerdo / disagree	La vista	Sensacion	No lo se	Despues
87	Si / Yes	No	24	Mujer / Female	Argentina	de acuerdo / agree	de acuerdo / agree	de acuerdo / agree	de acuerdo / agree	de acuerdo / agree	de acuerdo / agree	de acuerdo / agree	de acuerdo / agree	Taxto real	Que hay pocos simuladores	Con mas teoria acerca de los conductos y limas	Amtes simulador despues diente
89	Si / Yes	No	20	Mujer / Female	España	de acuerdo / agree	de acuerdo / agree	completamente de acuerdo / completely agree	neutral	neutral	neutral	neutral		Ver lo que vas a hacer Antes de ponerla en	Que va muy ràpido	No se	Antes

														practica			
90	Si / Yes	No	20	Mujer / Female	España	neutral	neutral	neutral	neutral	neutral	neutral	neutral	neutral	Es realista	Que no regula bien la fuerza	nidea	antes
91	Si / Yes	No	23	Hombre / Male	france	de acuerdo / agree	de acuerdo / agree	completamente de acuerdo / completely agree	de acuerdo / agree	de acuerdo / agree	de acuerdo / agree	de acuerdo / agree	de acuerdo / agree	podemos sentir la textura de los diferentes tejidos dentales	la vision	mejorar el manejo de instrumentos	antes
92	No	No	23	Hombre / Male	francia	en desacuerdo / disagree	en desacuerdo / disagree	en desacuerdo / disagree	en desacuerdo / disagree	en desacuerdo / disagree	en desacuerdo / disagree	en desacuerdo / disagree	en desacuerdo / disagree	no se	no hay	no se	después
93	Si / Yes	No	25	Hombre / Male	Italy	neutral	neutral	neutral	neutral	neutral	neutral	neutral	neutral	Entrenar sobre sensacion real	Se hace poco	Con mas practica	Antes
94	Si / Yes	No	22	Hombre / Male	Francia	en desacuerdo / disagree	de acuerdo / agree	en desacuerdo / disagree	en desacuerdo / disagree	neutral	completamente en desacuerdo / completely disagree	en desacuerdo / disagree	en desacuerdo / disagree	Menos estrés, permite entrenarse sin tener miedo de las consecuencias de un fallo	La utilización del espejo así como la sensación táctil	No lo sé	Antes para entrenarme
95	Si / Yes	No	25	Hombre / Male	Italia	de acuerdo / agree	de acuerdo / agree	completamente de acuerdo / completely agree	de acuerdo / agree	completamente de acuerdo / completely agree	de acuerdo / agree	de acuerdo / agree	completamente de acuerdo / completely agree	Visualización práctica	Sensibilidad		Antes

96	Si / Yes	No	25	Hombre / Male	Francia	completamente en desacuerdo / completely disagree	completamente en desacuerdo / completely disagree	completamente en desacuerdo / completely disagree	completamente en desacuerdo / completely disagree	completamente en desacuerdo / completely disagree	completamente en desacuerdo / completely disagree	completamente en desacuerdo / completely disagree	completamente en desacuerdo / completely disagree	Realisto	Ninguna	Con tecnologia	Antes
97	Si / Yes	No	24	Mujer / Female	France	neutral	de acuerdo / agree	de acuerdo / agree	de acuerdo / agree	en desacuerdo / disagree	neutral	de acuerdo / agree	en desacuerdo / disagree	sentir mas la textura de los tejidos	no hay	explicar mas lo ancho que tiene que quedar la cavidad	antes
101	Si / Yes	No	21	Hombre / Male	Italia	neutral	neutral	neutral	neutral	neutral	neutral	neutral	neutral	La práctica	No ser vivo	Más prácticas	Antes
102	Si / Yes	No	21	Mujer / Female	02/05/2003	completamente en desacuerdo / completely disagree	completamente en desacuerdo / completely disagree	completamente en desacuerdo / completely disagree	completamente en desacuerdo / completely disagree	completamente en desacuerdo / completely disagree	completamente en desacuerdo / completely disagree	completamente en desacuerdo / completely disagree	completamente en desacuerdo / completely disagree	To train infinity	The dolor of the head	More teeth	Antes
105	Si / Yes	Sí / Yes	23	Mujer / Female	Francia	neutral	de acuerdo / agree	en desacuerdo / disagree	en desacuerdo / disagree	en desacuerdo / disagree	neutral	en desacuerdo / disagree	completamente en desacuerdo / completely disagree	Do this when we are beginning in 1er year would be better	Its not the same sensation	Just practice in real is better	Before
106	No	Sí / Yes	19	Mujer / Female	Fr	de acuerdo / agree	de acuerdo / agree	de acuerdo / agree	de acuerdo / agree	de acuerdo / agree	de acuerdo / agree	de acuerdo / agree	de acuerdo / agree	Si	Si	Si	Si
108	Si / Yes	No	26	Mujer / Female	France	neutral	de acuerdo / agree	en desacuerdo / disagree	de acuerdo / agree	en desacuerdo / disagree	de acuerdo / agree	de acuerdo / agree	completamente en desacuerdo / completely disagree	It allow us to understand better the procedure	That is not the same thing that in real life	More practice	Before

109	Si / Yes	No	24	Mujer / Female	France	de acuerdo / agree	de acuerdo / agree	de acuerdo / agree	de acuerdo / agree	en desacuerdo / disagree	de acuerdo / agree	de acuerdo / agree	en desacuerdo / disagree	Simulating real life situations	Can turn around the mouth (not realistic)	Someone showing us before hand how to do	Before
111	Si / Yes	No	20	Hombre / Male	France	neutral	de acuerdo / agree	neutral	completamente de acuerdo / completely agree	neutral	de acuerdo / agree	neutral	de acuerdo / agree	Practice	Reproduction and précision	Increase the Space accuracy of the instruments	Before
113	Si / Yes	No	22	Mujer / Female	France	en desacuerdo / disagree	completamente de acuerdo / completely agree	de acuerdo / agree	de acuerdo / agree	neutral	de acuerdo / agree	de acuerdo / agree	de acuerdo / agree	The fait that we can restart over and over	the view	i dont know	before
114	Si / Yes	No	20	Mujer / Female	francia	completamente de acuerdo / completely agree	completamente de acuerdo / completely agree	completamente de acuerdo / completely agree	completamente de acuerdo / completely agree	completamente de acuerdo / completely agree	completamente de acuerdo / completely agree	completamente de acuerdo / completely agree	completamente de acuerdo / completely agree	training an other sensation	simulation	more explication and démonstration	yes before
115	Si / Yes	No	21	Mujer / Female	francia	neutral	de acuerdo / agree	de acuerdo / agree	de acuerdo / agree	en desacuerdo / disagree	completamente en desacuerdo / completely disagree	de acuerdo / agree	en desacuerdo / disagree	visualises	practice	mas practicas	yes
116	Si / Yes	No	23	Mujer / Female	France	de acuerdo / agree	de acuerdo / agree	neutral	neutral	de acuerdo / agree	de acuerdo / agree	de acuerdo / agree	neutral	We can train on a lot of different things	We don’t have the test of the mouth and the position		Yes

															isn't the same as a real mouth		
119	Si / Yes	No	21	Mujer / Female	Españs	de acuerdo / agree	de acuerdo / agree	completamente de acuerdo / completely agree	completamente de acuerdo / completely agree	en desacuerdo / disagree	de acuerdo / agree	de acuerdo / agree	en desacuerdo / disagree	Aprender a abrir la cámara pulpar	La apertura		Antes
120	Si / Yes	No	21	Mujer / Female	España	de acuerdo / agree	de acuerdo / agree	de acuerdo / agree	de acuerdo / agree	de acuerdo / agree	de acuerdo / agree	de acuerdo / agree	de acuerdo / agree	Que podemos repetir tantas veces como queramos	Que no es real	En simulador	Antes
121	Si / Yes	No	20 años	Mujer / Female	España	completamente de acuerdo / completely agree	completamente de acuerdo / completely agree	completamente de acuerdo / completely agree	completamente de acuerdo / completely agree	de acuerdo / agree	completamente de acuerdo / completely agree	completamente de acuerdo / completely agree	en desacuerdo / disagree	El poder tener esa sensación táctil real de los tejidos del diente que no nos permite tener un diente de resina.	El hecho de ser un aparato con un solo diente y no una cabeza en la que tienes que saber posicionarte y tienes más obstáculos para poder realizar el tratamiento, lo que simula una persona real.	Poder practicar con el Simodont y con los dientes de resina creo que está muy bien, quizás como un extra también sería interesante ver vídeos o	Antes

																fotos de tratamientos reales.	
122	Si / Yes	No	21	Mujer / Female	francia	de acuerdo / agree	completamente de acuerdo / completely agree	completamente de acuerdo / completely agree	completamente de acuerdo / completely agree	en desacuerdo / disagree	completamente de acuerdo / completely agree	completamente de acuerdo / completely agree	de acuerdo / agree	Nos permite sentir la textura del diente.	El diente está solo, lo podemos girar en todas direcciones lo cual no es la realidad	no sé	despues
123	Si / Yes	No	24	Hombre / Male	France	de acuerdo / agree	de acuerdo / agree	de acuerdo / agree	de acuerdo / agree	de acuerdo / agree	de acuerdo / agree	de acuerdo / agree	de acuerdo / agree	Mejorarse Practicar más	No hay	No lo sé	Antes es mejor
125	Si / Yes	Sí / Yes	22	Mujer / Female	Francia	neutral	de acuerdo / agree	neutral	de acuerdo / agree	de acuerdo / agree	neutral	neutral	neutral	Repetir Procedimientos tantas veces como sea necesario.	Los costes y los fallos.	A lo mejor con un simulador.	Prefiero el uso del simulador antes de la práctica con dientes de resina.
126	Si / Yes	No	21	Mujer / Female	España	neutral	de acuerdo / agree	de acuerdo / agree	de acuerdo / agree	neutral	neutral	de acuerdo / agree	en desacuerdo / disagree	Obtener mejores habilidades manuales.	No es igual que trabajar con los dientes de resina ya que no pode-	Mejorar la cucharilla	Antes

															mos movernos igual.		
127	Si / Yes	No	20	Hombre / Male	Francia	en desacuerdo / disagree	en desacuerdo / disagree	neutral	neutral	en desacuerdo / disagree	neutral	neutral	completamente en desacuerdo / completely disagree	Descubrir un poco, pero no es posible de aprender sobre el	Falta de una explicación o demonstracion antes de hacerlo	Mejorar el simodont, poner potencialmente una video demostrativa	antes
128	Si / Yes	No	21	Mujer / Female	Francia	completamente de acuerdo / completely agree	completamente de acuerdo / completely agree	completamente de acuerdo / completely agree	completamente de acuerdo / completely agree	completamente de acuerdo / completely agree	completamente de acuerdo / completely agree	completamente de acuerdo / completely agree	completamente de acuerdo / completely agree	Poder entrenarse	A veces no vemos bien	Poder amplificar el tamaño un poco más	Antes
129	Si / Yes	No	22	Mujer / Female	Francia	en desacuerdo / disagree	de acuerdo / agree	completamente de acuerdo / completely agree	en desacuerdo / disagree	en desacuerdo / disagree	en desacuerdo / disagree	en desacuerdo / disagree	completamente en desacuerdo / completely disagree	Sentir las texturas de esmalte y dentina	Explicación, tutorial	Hacer una práctica de exploración del símodont y explicación de cómo se hacen los tratamientos	Simodont antes de practicar en diente

130	Si / Yes	No	20	Mujer / Female	Francia	de acuerdo / agree	de acuerdo / agree	completamente de acuerdo / completely agree	de acuerdo / agree	de acuerdo / agree	neutral	de acuerdo / agree	de acuerdo / agree	Es que te permite aprender y equivocarte sin consecuencias reales. Puede repetir situaciones una y otra vez hasta perfeccionarla	La falta de realismo total, nunca puede replicar a 100% las condiciones físicas, emocionales y ambientales	Mejorar la formación a traves de simuladores avanzados	Preferia tener la practica de pulpotomia antes porque no tenemos presión y una mayor comprensión tridimensional (entender mejor la anatomía del diente)
133	Si / Yes	No	24	Hombre / Male	France	de acuerdo / agree	de acuerdo / agree	de acuerdo / agree	de acuerdo / agree	de acuerdo / agree	de acuerdo / agree	de acuerdo / agree	de acuerdo / agree	Practicar	Para pulepectomia	No atraviészar el diente	Antes
134	Si / Yes	No	22	Mujer / Female	Francia	en desacuerdo / disagree	completamente de acuerdo / completely agree	completamente de acuerdo / completely agree	completamente de acuerdo / completely agree	neutral	neutral	en desacuerdo / disagree	completamente en desacuerdo / completely disagree	Que hacemos une cavidad demás	No se exactemente pero soy más cómoda con los fantômas		Después
135	Si / Yes	No	23	Mujer / Female	España	completamente de acuerdo / completely agree	de acuerdo / agree	de acuerdo / agree	de acuerdo / agree	de acuerdo / agree	de acuerdo / agree	de acuerdo / agree	de acuerdo / agree	Sentir en primera mano como sería hacer una	No es muy sensible	Dar más tiempo a los alumnos	Antes

													pulpotomía antes de hacerlo con un paciente real			
136	Si / Yes	Sí / Yes	22	Mujer / Female	France	completamente en desacuerdo / completely disagree	neutral	de acuerdo / agree	de acuerdo / agree	neutral	en desacuerdo / disagree	en desacuerdo / disagree	completamente en desacuerdo / completely disagree	Que hay una diferencia de textura entre el esmalte y la dentina, o cuando hay tejido cariado que se siente y se ve con la color aunque con las dientes de resina hacemos con la imaginación para quitar tejido cariado	Que se puede rotar los modelos en todos los lados aunque en la vida real o en en fantôme estamos en la misma position que en la que vamos a trabajar	Explicar antes nosotros lo que debemos ver, las texturas, cómo estar mas conservador …	Después
137	Si / Yes	No	20	Mujer / Female	España	completamente de acuerdo /	completamente de acuerdo /	completamente de acuerdo /	completamente de acuerdo /	completamente de acuerdo /	completamente de acuerdo /	completamente de acuerdo /	completamente de acuerdo /	Me parece una	creo que debe ser algo	Hacer más, no	En dientes de resina

	completely agree	completely agree	completely agree	completely agree	completely agree	completely agree	completely agree	completely agree	buena forma de practicar y creo que nos acercamos más a la realidad a la hora de utilizar el material rotatorio	complementario a las prácticas con muñecos.	solo una
