# Supplementary material for: Can haptic reinforced VR simulation transform preclinical pulpotomy training? Insights into skill acquisition, student perceptions, and educational impact: randomized controlled trial
Source: Front Oral Health. 2025 Sep 24;6:1677056. doi: 10.3389/froh.2025.1677056 (PMC12504311; doi:10.3389/froh.2025.1677056)
Supplement: Supplementary file 3 [file Table1.docx]

**Supplementary Table S1.**

Student perception questionnaire on pre-clinical haptic virtual reality simulation (HVRS) training. The table lists the closed-ended items rated on a 5-point Likert scale (1 = completely disagree; 2 = disagree; 3 = neutral; 4 = agree; 5 = completely agree) and the open-ended questions used to collect qualitative feedback.

| **Closed-ended questions**   1. Pulpotomy demonstration on the simulator allowed me to clearly comprehend the tasks expected from me. 2. Images of the teeth, pulp chamber, and instruments displayed on the simulator monitor looked realistic. 3. I could differentiate between the texture and hardness of enamel and dentine in the simulator device. 4. Tactile force feedback given by the simulator felt realistic. 5. Deroofing the pulp chamber on the simulator device felt similar to that on plastic teeth mounted on mannequins. 6. Training on the simulator device improved my fine motor dental skills. 7. Training on the simulator device improved my confidence in performing the pulpotomy procedure. 8. The simulator can replace conventional pre-clinical training on plastic teeth for the pulpotomy procedure. |
| --- |
| **Open-ended questions**   1. In your opinion what is the main benefit of simulator training? 2. In your opinion what is the main limitation of simulator training? 3. How could pre-clinical training for pulpotomy in primary teeth be improved in the future? 4. Would you prefer to have simulator training for primary molar pulpotomy before or after training on plastic tooth models? |
